# Contribution of Magnetic Resonance Imaging Studies to the Understanding of Cerebral Malaria Pathogenesis

**DOI:** 10.3390/pathogens13121042

**Published:** 2024-11-27

**Authors:** Alicia Comino Garcia-Munoz, Isabelle Varlet, Georges Emile Grau, Teodora-Adriana Perles-Barbacaru, Angèle Viola

**Affiliations:** 1 Centre de Résonance Magnétique Biologique et Médicale (CRMBM) UMR 7339, Faculté des Sciences Médicales et Paramédicales la Timone, Aix-Marseille Université, CNRS, 13055 Marseille, France; alicia.comino-garcia-munoz@univ-amu.fr (A.C.G.-M.); isabelle.varlet@univ-amu.fr (I.V.); teodora.perles-barbacaru@univ-amu.fr (T.-A.P.-B.); 2 Vascular Immunology Unit, School of Medical Sciences, Faculty of Medicine, The University of Sydney, Medical Foundation Building (K25), Camperdown, NSW 2042, Australia; georges.grau@sydney.edu.au

**Keywords:** murine cerebral malaria, human cerebral malaria, pathogenesis, magnetic resonance imaging, magnetic resonance spectroscopy

## Abstract

Cerebral malaria (CM), the most lethal clinical syndrome of *Plasmodium falciparum* infection, mostly affects children under 5 in sub-Saharan Africa. CM is characterized by seizures and impaired consciousness that lead to death in 15–20% of cases if treated quickly, but it is completely fatal when untreated. Brain magnetic resonance imaging (MRI) is an invaluable source of information on the pathophysiology of brain damage, but, due to limited access to scanners in endemic regions, only until very recently have case reports of CM patients studied with advanced MRI methods been published. The murine model of experimental cerebral malaria (ECM) shares many common features with the human disease and has been extensively used to study the pathogenic mechanisms of the neurological syndrome. In vivo MRI studies on this model, the first of which was published in 2005, have contributed to a better understanding of brain lesion formation in CM and identified disease markers that were confirmed by MRI studies published from 2013 onwards in pediatric patients from endemic areas. In this review, we recapitulate the main findings and critically discuss the contributions of MRI studies in the ECM model to the understanding of human CM.

## 1. Introduction

Malaria is the worst parasitic disease in the world, with 249 million clinical cases in 2022 and an estimated annual death toll of approximately 600,000. It is a major public health problem worldwide with 85 malaria endemic countries and nearly half the world’s population at risk for Plasmodium infection [1]. Cerebral malaria (CM) is one of the most lethal clinical syndromes of *Plasmodium falciparum* infection [2,3], affecting mostly children under 5 in sub-Saharan Africa [3].

CM is characterized by seizures and impaired consciousness, leading to death in 15-20% of cases if it is quickly treated, but it is completely fatal when untreated [4]. In the pediatric population, CM begins with fever, vomiting and convulsions, rapidly followed by coma. Other neurological manifestations include signs of upper motor neuron lesions (increased muscle tone, brisk tendon reflexes, among others), abnormalities of brainstem reflexes such as oculocephalic, oculovestibular and papillary reflexes and retinopathy [5,6]. It is now well established that most patients present with brain swelling, which is considered a major predictor of death in children [7]. Clinically, pediatric CM is characterized by a higher incidence of convulsions, high cerebrospinal fluid (CSF) pressure and neurocognitive deficits, and, in contrast, shows a lower incidence of liver and renal failure compared to adult CM [8,9,10]. Although children account for the vast majority of CM deaths, the fatality rate in adults is higher than in children. In addition, seizures are rare in adults, as are abnormal brainstem reflexes, and brain swelling is not always predictive of death [9]. Differences in severity with age may be multifactorial, with an immunological component linked to the exposure and immaturity of the immune system in children but also with a greater vulnerability of the immature brain of young children [11].

There are no clear epidemiological data on whether the severity and mortality of CM are higher in men or women. However, a study in Indian patients revealed higher plasma levels of the pro-inflammatory chemokine CXCL10 in male subjects, linked to CXCL10 gene promoter polymorphism -1447A>G and associated with a greater susceptibility to CM in male patients [12].

Although CM as a clinical syndrome cannot be treated, the malaria and parasitemia can be treated by antimalaria drugs [13] but often remain ineffective against CM when administered at an advanced stage. Most patients succumb to the disease within a few hours after admission to hospital, despite the administration of antimalarial drugs. Even with the administration of artesunate, the current first-line treatment against severe malaria, the fatality rate is still 18% in children, 30% in adults and rises up to 50% in pregnant women [14]. Moreover, adjunctive therapies such as mannitol, used to reduce edema and decrease intracranial pressure, have proven inefficient or harmful [3,15]. The absence of medical facilities or the low quality of the diagnostic techniques for the identification of the parasite in blood smears in some sub-Saharan Africa endemic areas often delay early treatment [3]. Only very few vaccines have made it to the clinical trial stage. Although the RTS,S vaccine approved in 2021 is a great step towards preventing the death of many children, its deployment has several limitations, such as a limited supply in endemic countries, low effectiveness (30%) for severe malaria cases, a requirement of up to three doses and a booster dose and a rapid decline of the immune protection [16,17]. The R21/Matrix-M vaccine, introduced in 2023, requires the same number of doses, but its effectiveness is 65% for CM 18 months after the first dose [18].

Some aspects of the pathogenesis of CM are still unclear. Although the *P. falciparum* does not penetrate the brain parenchyma [19], it is capable of disrupting the blood–brain barrier (BBB). The permeabilization of the BBB results from the sequestration, aggregation and adherence of parasitized erythrocytes, leukocytes and platelets to microvascular endothelial cells, leading to their activation. Thrombocytopenia is a common finding in *P. falciparum* infection, and while its pathogenesis remains unclear, it has been associated with a poor outcome and platelets have been identified as a key contributor to the endothelial alterations [20,21,22]. Moreover, the overproduction of microvesicles (previously called microparticles) by platelets and endothelial cells would contribute to the pathogenesis of CM via the activation of coagulation and/or induction of inflammatory processes [21]. A dysregulated and harmful response of the host immune system, involving pro-inflammatory cytokines such as TNF, IFN-γ, IL-1, IL-6 and IL-12 [23], chemokines, adhesion molecules (ICAM-1, VCAM-1, E-selectin,) and effector cells (CD4^+^ and CD8^+^ T cells) [21,24,25] would determine the outcome of CM.

Neuroimaging studies in human CM (HCM) have been limited until recently. Computed tomography (CT) scans have been shown to underestimate the extent of disease, with a normal CT scan being a common finding [26]. A few magnetic resonance imaging (MRI) reports in CM patients—explored in the acute or the recovery phase—have described diverse anomalies on T_1_-weighted imaging (T_1_w), consisting in increased brain volume, diffuse cerebral edema and herniation through the foramen magnum associated with either ventriculomegaly or the crushing of the lateral ventricles [7,27,28,29]. Parenchymal lesions detected on T_2_-weighted imaging (T_2_w) consisted in focal hyperintensities in several white matter tracts including the corpus callosum, and were considered as indicative of vasogenic edema, gliosis or cell death [30,31]. T_2_*-weighted imaging (T_2_*w) and susceptibility-weighted imaging (SWI) detected focal hemorrhages (petechia), a hallmark of the neurological syndrome, and infarction in some patients at the cortical and subcortical levels [29,30,32,33].

Although brain MRI is an invaluable source of information on the pathophysiology of brain damage in HCM, so far, only a few CM patients have been studied with advanced MRI methods that can provide functional parameters such as perfusion in addition to morphological and microstructural information. Similarly, only one case study [34] exploits magnetic resonance spectroscopy (MRS) capable of assessing metabolic changes. In most endemic regions, mostly low- to medium-income countries in sub-Saharan Africa [35], access to MRI scanners is non-existent or limited to low-field magnets, which are useful for recognizing CM but may not provide mechanistic information on disease evolution and neuropathology development.

**Figure 1:**
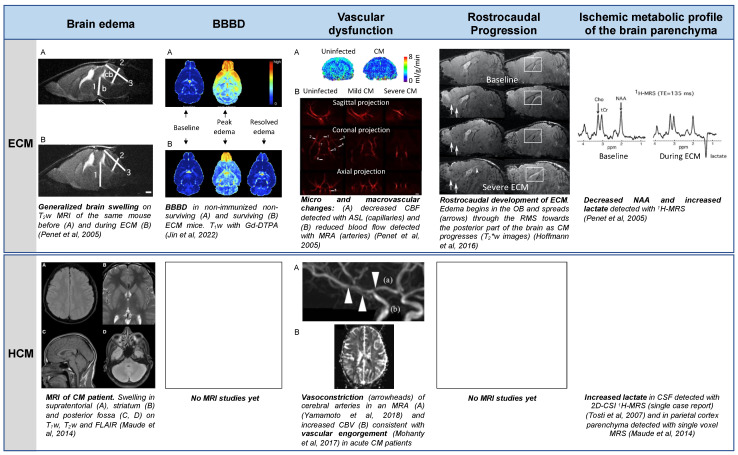
A summary of the main contributions of MRI and MRS to the understanding of the pathogenesis of CM. Some characteristics, like brain swelling or vascular changes, have been found using similar imaging techniques in ECM and HCM. Other characteristics, like BBBD, the direction of disease progression or the metabolic profile have been shown in ECM, but have not yet been fully explored in HCM. Abbreviations: ^1^H-MRS, proton magnetic resonance spectroscopy; 2D-CSI, two-dimensional chemical shift imaging; ASL, arterial spin labeling; BBBD, blood–brain barrier disruption; CBF, cerebral blood flow; CBV, cerebral blood volume; Cho, choline-containing compounds; CM, cerebral malaria; CSF, cerebrospinal fluid; ECM, experimental CM; FLAIR, fluid attenuated inversion recovery MRI contrast; Gd-DTPA, gadopentetic acid; MRA, magnetic resonance angiography; MRI, magnetic resonance imaging; NAA, *N*-acetylaspartate; OB, olfactory bulb; ppm, parts per million; RMS, rostral migratory stream; T_1_w, T_1_-weighted MRI contrast; T_2_w, T_2_-weighted MRI contrast; T_2_*w, T_2_*-weighted MRI contrast; tCr, total creatine; TE, time of echo. Adapted from [28,34,41,42,43,44]. All presented data are CC-BY 4.0. Adapted from [39], copyright [2005] Society for Neuroscience.

Experimental cerebral malaria (ECM) in mice has been extensively used to study the pathogenic mechanisms of the disease and is regarded as a good model of HCM, as it shares many common features with the human disease [36,37,38] despite some differences. In vivo MRI studies of this model, the first of which was published in 2005 [39], have contributed to a better understanding of brain lesion formation in CM and identified disease markers shared by the human disease, as confirmed by studies published from 2013 onwards on pediatric populations [7,27,29,40]. In this review, we recapitulate the findings and advances resulting from the MRI study of the ECM model (Figure 1) and critically discuss their relevance to the human disease.

## 2. The Murine Model of CM

The ECM murine model is the best characterized and most widely used model of CM. It allows the investigation of disease pathogenesis and has been successfully applied in the antimalarial drug discovery process with the identification of major antimalarial drugs, notably mefloquine, halofantrine and artemisinin derivatives [14,45]. CM models have also been developed in non-human primates (NHP), but the cost and ethical constraints of obtaining large cohorts of these subjects have prevented extensive research on them [46]. In addition, NHP models show little similarity with the human disease, as they present no significant MRI changes in the brain after disease induction [47].

The ECM mouse model consists in the infection of CM-susceptible mouse strains (e.g., C57BL/6, CBA/J SLJ/J, 129/Ola, Swiss or NMRI) with murine Plasmodium strains such as *P. berghei* ANKA (PbA), *P. berghei* K173 or *P. yoelii* XL [48,49,50]. The host–parasite combination PbA/CBA/J or PbA/C57BL/6 is the best characterized and most widely used model [38,48,51]. These mice share clinical and neurological signs with human CM, including ataxia, seizures, respiratory distress, loss of consciousness, coma and death within a few days after infestation in the absence of treatment [36,39]. The rapid disease progression is therefore a common feature with the human disease [13]. CM-resistant mouse strains have no apparent neurological signs and die of severe anemia, weeks after inoculation. As in humans, the development of the cerebral syndrome seems to be governed by an age-dependent immune response. Indeed, in young CM-susceptible mice, higher levels of pro-inflammatory cytokines were measured after PbA inoculation compared to middle-aged mice. In addition, older mice that had not been exposed to the parasite showed an increase in CD4^+^CD25^+^Foxp3^+^ regulatory T-cells and IL-10 after infection, thus preventing CM [52]. As for the effect of sex on the development of CM, sexual steroids appear to play a role in the regulation of the expression of pro-inflammatory cytokines in the mouse brain, resulting in higher levels in ECM males in most brain areas [53]. In HCM, polymorphisms in the gene promoter sequence of CXCL10, a chemokine induced by TNF and IFN-γ, might be partially responsible for the difference in susceptibility to CM of males [51].

There is still a lack of consensus on many critical aspects of the pathophysiology of HCM, since, until recently, most knowledge has relied on the analysis of post-mortem brains [36]. The ECM model has proven useful for elucidating unclear immunological mechanisms in HCM [54], such as the activation and accumulation of platelets, monocytes and NK cells in the brain microvasculature, a feature first discovered in ECM [36,55,56] and shared with HCM [10]. Other features, such as the upregulation of endothelial cell adhesion molecules [25,57], the role of microvessels in immunopathogenesis [21] or the reduced expression of endothelial tight junction proteins involved in maintaining BBB integrity [58,59] are also common to human and murine diseases.

The relevance of the murine model has been the subject of controversy [60], as it shows little adhesion of parasitized red blood cells (pRBCs)—although it includes platelets [61]—to the vascular endothelium of the brain vessels, a characteristic feature of HCM that is considered the primary cause of coma and death [36,62]. Platelet sequestration is actually a major pathogenic element in both HCM and ECM [63,64]. However, it has already been proven that a single pRBC is capable of obstructing a brain capillary in mice and that erythrocyte sequestration is part of the pathogenesis of ECM [21,65,66]. Furthermore, the sequestration of pRBCs is not always present in HCM, so there are certainly other pathogenic features and causes of death, especially in children [60,62].

## 3. MRI and MRS Findings in ECM

Before MRI characterization of the mouse model, the MRI data available for HCM were limited to single cases, which did not help to advance the understanding of the pathogenesis of the disease and led to discrepancies in the discoveries. However, many features observed in HCM, especially once MRI studies on large cohorts were conducted in endemic countries [27], had already been shown on the model as early as 2005 [39] and have been further confirmed by other studies (see Table 1 and Table 2 and Supplementary Tables S1 and S2). Penet et al. [39] were the first to characterize the pathophysiology of ECM using in vivo MRI and MRS techniques.

### 3.1. Brain Edema

One of the main characteristics of ECM is the presence of brain and ventricle swelling [39], as seen by anatomical and diffusion weighted imaging (DWI) MRI. The severity of this edema correlates with the severity of the disease [2], and its location varies as the disease progresses. At day 5-6 post infection, it can be found as a T_2_w hyperintensity in the white matter of the corpus callosum or in the striatum [13]. In the advanced stages of the disease, the edema causes the protrusion of the brainstem into the foramen magnum, which eventually leads to coma and death [39,67].

The nature of this edema can be determined by the changes in the apparent diffusion coefficient (ADC) evaluated with DWI. The increase in ADC observed in ECM [42] is indicative of vasogenic edema caused by the leakage of fluids from the capillaries due to the disruption of the blood–brain barrier (BBBD) [71]. Other studies observed a decrease in ADC [39], indicative of cytotoxic edema due acute ischemia. This ischemia causes a failure of the Na+/K+ pump leading to an accumulation of water in the intracellular space [81]. Penet et al. [39] detected ischemia both by perfusion MRI with the arterial spin labeling (ASL) technique and by ^1^H-MRS with the detection of high levels of brain lactate. Their study confirmed microcapillary dysfunction, but also highlighted the previously unidentified damage to arteries caused by vasogenic edema. An interesting result reported in Mohanty et al. [44] is the observation of regional differences in vulnerability to the two types of edema, probably linked to each region’s microvascularization.

The presence of brain edema in HCM has been controversial for many years and has only recently been accepted [7,27,30,32]. Increased brain volume and raised intracranial pressure have been linked to the onset of neurological and cognitive symptoms, as well as to a higher case fatality risk [7,28].

Moreover, in humans, the edema expresses a differential pattern with age. In pediatric CM, edema is severe [7,40] and, just as in the mouse model, leads to death due to the herniation of the brainstem [71]. In adults, brain swelling is milder and not directly associated with mortality. The most probable cause of death in adults with CM is severe brain hypoxia following the mechanical obstruction of capillaries by pRBCs and immune cells in the context of multiorgan failure [28,71].

**Figure 2:**
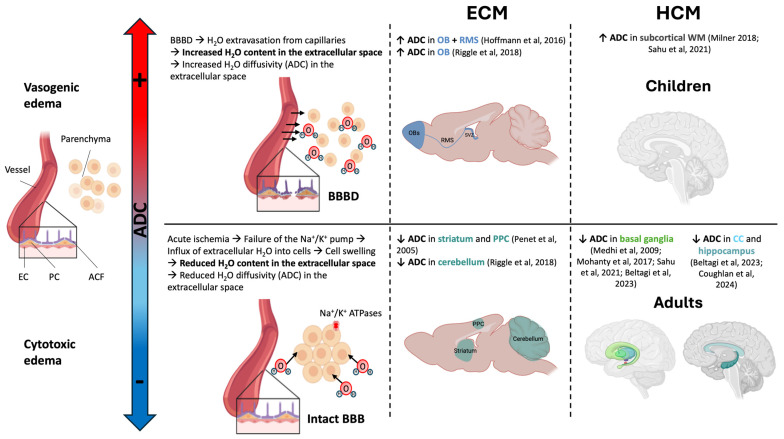
Two different mechanisms of edema can be distinguished using the ADC in ECM and HCM. Vasogenic edema is characterized by a rise in ADC, as there is an increase in the water content in the extracellular space, eventually causing brain swelling. Cytotoxic edema is characterized by a decrease in ADC because the ischemia leads to reduced extracellular space due to the free water entering the brain cells. Both mechanisms can be present at the same time in CM depending on the affected structure or the age of the patient in the case of HCM. Abbreviations: ACF, astrocyte endfeet; ADC, apparent diffusion coefficient; BBBD, blood–brain barrier disruption; CC, corpus callosum; EC, endothelial cell; ECM, experimental cerebral malaria; HCM, human cerebral malaria; OB, olfactory bulb; PC, pericyte; PPC, posterior parietal cortex; RMS, rostral migratory stream; SVZ, subventricular zone; WM, white matter. Image created using image database Biorender [13,29,32,39,42,44,71,74,76].

Brain edema is predominantly vasogenic in acute pediatric CM, with raised ADC values and T_2_ hyperintensity in the white matter (WM) [29]. However, decreased ADC has been found in multiple loci in children in a recent study [75]. In adult CM, it has been characterized as cytotoxic with diffusion restriction, as evidenced by ADC decrease due to severe hypoxia [30,71,74,80]. A study in a mixed cohort of children and adults [44] recently found that both mechanisms of edema can happen at the same time in HCM, in different parts of the brain. The authors conclude that in CM there might be a “multifactorial brain swelling” [44], which could explain the findings of the two types of edema both in the mouse model [13,39] and in human patients [28,75,76] (Figure 2).

### 3.2. Blood Brain Barrier Disruption

The presence of a vasogenic edema in the ECM model is preceded by a predominantly rostral BBBD observed with the use of gadolinium-based contrast agents (GBCA) [82]. The BBBD was first described using the intravenous injection of gadopentetic acid [39]; it has been confirmed by many other studies [2,13,41,42,68]. This BBBD occurs on days 6–7 after the infection and is also accompanied by a blood–CSF barrier disruption [39,41]. Furthermore, in the areas where the BBBD is more pronounced, like the olfactory bulb (OB), there is a higher amount of microhemorrhages both during the edema and after its reversal with treatment. The amount and intensity of these microhemorrhages might be an indication of the severity of the cerebral syndrome at the peak of parasitemia [41].

Unlike in ECM, there are no MRI studies that show the BBBD in humans. GBCA are costly or inaccessible in endemic areas, and they should be avoided in pediatric patients and those with kidney failure, as reviewed in [83]. We found no evidence of other MRI techniques assessing BBBD in CM patients. However, immunohistochemistry studies in post-mortem HCM brain have shown a possible failure of microvascular endothelial cells because of an upregulation of cell adhesion molecules like ICAM-1 and a reduction in the expression of cell junction proteins, along with the activation of astrocytes and glial cells after Plasmodium infection in HCM [36,84,85].

### 3.3. Vascular Dysfunction

The ECM murine model also allowed the characterization of the vascular component of the disease using perfusion MRI with arterial spin labeling (ASL) and magnetic resonance angiography (MRA). The compression of the arteries [39] and an affected microvasculature is evidenced by reduced cerebral blood flow (CBF), especially in the striatum and the cortex [39,70] in infected mice. The vasoconstriction of the cerebral arteries has been reported in individual cases in HCM using MRA [43].

### 3.4. Spatiotemporal Development of the Disease

Studies from Zhao et al. [69] and Hoffmann et al. [42] identified the OBs as the starting point of ECM. Before any clinical signs occur, the edema shows as multifocal hyperintense regions in the OBs on T_1_w images, followed by hypointense lesions on T_2_*w images that correspond to microhemorrhages [42]. The edema and the BBBD start in this region and spread caudally along the rostral migratory stream, the dorsal migratory stream and eventually reach the brainstem, which is crushed [39,71]. The OBs possess a distinct microvasculature of trabecular small capillaries that are easily occluded by pRBCs, causing lesions and microhemorrhages [69]. The BBB in the OB also has a higher permeability, even in the absence of pathology and a large population of antigen presenting cells like macrophages or microglia [86]. All this could help to explain why this brain structure is particularly vulnerable and might be the starting point of the inflammation caused by the parasite moving into the brain via these specific microvessels [42].

Other structures that have been shown to be affected early on by the disease are the optical and trigeminal nerves, which appear hypointense in the T_2_w images as early as 4 or 5 days after the infection [67] and could help to identify possible early markers of the cerebral syndrome.

### 3.5. Metabolic Changes

Finally, magnetic resonance spectroscopy (MRS) has helped identify metabolic anomalies of CM. The main finding using this method in ECM is an obvious ischemic metabolic profile that supports the findings of ischemic lesions using MRI [39,87]. This metabolic profile includes an increase in brain lactate, alanine and glutamine, indicators of tissue hypoxia and anaerobic metabolism and a decrease in *N*-acetylaspartate (NAA) [39,70,87,88,89].

The reduction in NAA, a marker of axonal integrity and mitochondrial activity, is indicative of neuronal damage, which fits the pattern of ischemia [90]. Moreover, the levels of NAA have been positively correlated to alterations in the CBF in ECM, which suggests that the neuronal dysfunction might be related to a deficient oxygen delivery [70,90].

Despite lower numbers of MRS studies in HCM, this pattern of metabolic changes has also been found. HCM causes an increase in the lactate/creatine ratio in CSF as seen using two-dimensional chemical shift imaging (2D-CSI) [34], which decreases along with parasitemia, confirming the long-recognized role of lactate in CM pathogenesis [91].

Interestingly, one ex vivo MRS study on ECM showed that brain lactate was increased in females but not in male mice [88]. Sexual differences have also been observed in immunological studies using the ECM model [53,92], and although the demographic data in HCM are rarely separated by sex, some studies have reported a higher mortality, a higher incidence of convulsion and lower hemoglobin levels in nonpregnant females than in males [93]. So far, none of the MRI studies on HCM have analyzed their data in relation to sex.

## 4. Relevance of ECM and MRI/MRS Method

The ECM mouse model was able to help characterize many traits of CM that have later been confirmed in the human population once magnets with sufficient field strength became available in endemic areas. 

Considering the age specific patterns that HCM patients present [71] and the characteristics of the ECM model, we observe a greater similarity between the murine model and pediatric CM (in children under 5 years of age [3]). As in the mouse model, children with CM present a more severe edema, leading to the herniation of the brainstem [7,40,71]. This edema also tends to have a greater vasogenic component both in mice and in children [29,39,42]. The quantity of microhemorrhages at the peak of the disease would correlate with the risk of fatal outcome in both HCM and ECM [41].

As mentioned before, the drugs that have been used for decades in the treatment of HCM, like chloroquine or artesunate, have a moderate effectiveness and only when given as early as possible [14]. However, to date, there is no approved treatment for the advanced stage of CM [13]. This is a very common scenario in endemic areas, where people have less access to fast health care. The animal model of ECM also opened the possibility to conceive new therapeutic strategies and assess them using MRI. Riggle et al. [13] recently developed a treatment based on a glutamine antagonist that can rescue mice from ECM, even when major signs like BBBD and brain swelling have already appeared. To our knowledge, this is the first study assessing the efficacy of a treatment using MRI in ECM.

## 5. Conclusions and Future Directions

The ECM mouse model has proved to be a relevant model of HCM. Despite some criticism, immunology and imaging studies show very clear similarities between the murine and the human diseases.

The discoveries made with the ECM model have helped to reconcile controversies regarding the characteristics of CM such as the presence of edema, now known as a major determinant of disease progression. Since the first characterization of the model with MRI/MRS in 2005 [39], many features of the disease have been established in ECM using imaging techniques. Many of these characteristics have been confirmed years later in pediatric cohorts when MRI became available in certain sub-Saharan countries. Moreover, these discoveries and the techniques used in these studies have sparked the use of MRI as a research tool in HCM [94].

Further research using the ECM model will give insight into how the cerebral syndrome of the disease affects children under 5 years old, a population group that accounts for more than 60% of all the malaria deaths worldwide [3]. This will help to establish more efficient diagnostic tools and assess new therapies that might overcome the limitation of the current drugs to relieve the burden of the disease in endemic countries.

## Figures and Tables

**Table 1:** Summary of the main findings on ECM using MRI/MRS techniques.

PATHOGENIC FEATURE	FINDINGS	REFERENCES
**Brain swelling and vasogenic edema**	Increased brain volume	[2,39,41,67]
	Cerebellum crushing	[13,39,67]
	Brainstem engulfment	[39,67]
	Increased diffusion in OB, WM and RMS	[13,42]
**Cytotoxic edema** **(restricted diffusion)**	Striatum	[39]
	Parietal cortex	[39]
	Cerebellum	[13]
**Changes in ventricle volume**		[39]
**BBBD**	OB, CC, external capsule	[2,13,39,41,42,68]
**Blood–CSF disruption**		[2,13,39]
**Lesions**	WM	[39,67]
	Caudate putamen	[39,67]
	Brainstem	[67]
	OB	[13,42,67]
	Cranial nerves	[67]
**(Micro)hemorrhages**	Cerebrum	[39,67]
	Cerebellum, brainstem	[67]
	OB	[41,67,69]
**Vascular function**	Reduced blood flow in cerebral arteries	[39]
	Reduced CBF (capillaries)	[39,70]
	Vascular inflammation	[2]
**Neurometabolic changes**	Reduced NAA	[39,70]
	Increased Glx	[39]
	Decreased (PCr + β-ATP)/Pi	[39]
	Decrease in pH	[39]
	Increased lactate	[39]
**Development of the disease**	Rostrocaudal (via RMS to DMS)	[2,42]

Abbreviations: ATP, adenosine triphosphate; BBBD, blood–brain barrier disruption; CBF, cerebral blood flow; CC, corpus callosum; DMS, dorsal migratory stream; Glx, glutamate + glutamine; NAA, *N*-acetylaspartate; OB, olfactory bulb; PCr, phosphocreatine; Pi, inorganic phosphate; RMS, rostral migratory stream; WM, white matter.

**Table 2:** Summary of the main findings on HCM using MRI/MRS techniques.

PATHOGENIC FEATURE	FINDINGS			
	ADULT CM		PEDIATRIC CM	
**Brain edema**	Mild and diffuse	[28,32,41,43,71]	Severe	[10,27,40,41,71]
	Resolved in survivors	[71,72]	Resolved in survivors	[7,71]
			Uncal, cerebellum or brainstem herniation	[7,27,71]
			Associated with morbidity	[7,40]
			Persistent in survivors with neurological sequelae	[31,73]
**Vasogenic edema** **(increased diffusion)**	Cortex, posterior	[44]	Cortex	[27,44]
			Basal ganglia	[7,27]
			Corpus callosum	[7,27]
			Subcortical WM	[27,71]
			Posterior fossa	[7]
**Cytotoxic edema** **(restricted diffusion)**	Cortex	[28,74]	Cortex	[75]
	Basal ganglia	[32,44,71,74,76]	Basal ganglia	[29,44,75]
	Corpus callosum	[74,76]	Corpus callosum	[75,77]
	Thalamus	[33]	Subcortical WM	[29,71,75]
	Periaqueductal GM	[33]	Resolved in survivors	[71]
	Cerebellum	[33]		
	Brainstem	[33]		
	Hippocampus	[76]		
	Resolved in survivors	[71]		
**White matter** **lesions**	Focal	[78]	Persistent in survivors and correlating with long term neurological sequelae	[31,73,79]
	Periventricular WM	[30,80]	Periventricular WM	[27,73]
	Subcortical WM	[76,80]	Subcortical WM	[7,27,29,40,73]
	Corpus callosum	[28,30,74]	Corpus callosum	[27,29]
	Corona radiata	[33,76]		
**Cortical lesions**	Focal	[28]	Focal	[7,27,29,40]
			Persistent in survivors	[73]
**Subcortical lesions**	Thalamus	[30,32,33]	Thalamus	[7,27,40]
	Basal ganglia	[28,32,74]	Basal ganglia	[7,27,29,40,73]
	Cerebellum	[28,32,33]	Cerebellum	[27,73]
	Brainstem	[28,33]	Brainstem	[7,29,40,73]
	Hippocampus	[33]	Posterior fossa	[7,40]
**(Micro)hemorrhages**	Cortex	[78]		
	Basal ganglia	[41,74,76]	Basal ganglia	[41]
	Corpus callosum	[41,74,76]	Corpus callosum	[41]
	GM-WM junction	[41]	GM-WM junction	[41]
	Cerebellum	[41]	Cerebellum	[41]
	Brainstem	[33]		
	Thalamus	[33]		
	Frequency correlates with disease severity	[41]	Frequency correlates with disease severity	[41]
**Vascular function (congestion)**	Basal ganglia	[44]	Basal ganglia	[29,44]
	Reversible cerebral vasoconstriction syndrome	[43]		
**Neurometabolic changes**	Increased lactate/Cre in CSF	[34]		
	Increased lactate/Cre in parietal cortex	[28]		
	Increased Cho/Cre in parietal cortex	[28]		

Abbreviations: Cho: choline-containing compounds; Cre, creatine + phosphocreatine; CSF, cerebrospinal fluid; GM, gray matter; WM, white matter.

## Supplementary Materials

The following supporting information can be downloaded at: https://www.mdpi.com/article/10.3390/pathogens13121042/s1, Table S1: MRI/MRS studies of ECM; Table S2: MRI/MRS studies of HCM.

## Abbreviations

**ACF**	Astrocyte endfeet.
**ADC**	Apparent diffusion coefficient (of water).
**ASL**	Arterial spin labeling (perfusion MRI method without contrast agent injection).
**ATP**	Adenosine triphosphate.
**BBB**	Blood–brain barrier.
**BBBD**	Blood–brain barrier disruption.
**CBF**	Cerebral blood flow.
**CC**	Corpus callosum.
**Cho**	Choline-containing compounds.
**CM**	Cerebral malaria.
**Cr**	Creatine + phosphocreatine.
**CSF**	Cerebrospinal fluid.
**2D-CSI**	Two-dimensional chemical shift imaging (method combining MRI and MRS).
**DMS**	Dorsal migratory stream.
**DWI**	Diffusion weighted imaging.
**EC**	Endothelial cell.
**ECM**	Experimental cerebral malaria.
**FLAIR**	Fluid-attenuated inversion recovery (pulse sequence enabling the suppression of the signal from liquids such as CSF in brain).
**GBCA**	Gadolinium-based contrast agents.
**Gd-DTPA**	Gadopentetic acid (contrast agent).
**Glx**	Glutamine + glutamate.
**GM**	Gray matter.
**MRA**	Magnetic resonance angiography.
**MRI**	Magnetic resonance imaging.
**MRS**	Magnetic resonance spectroscopy.
**NAA**	*N*-acetylaspartate.
**OB**	Olfactory bulb.
**PbA**	*Plasmodium* berghei ANKA.
**PC**	Pericyte.
**PCr**	Phosphocreatine.
**PPC**	Posterior parietal cortex.
**ppm**	Parts per million.
**pRBC**	Parasitized red blood cell.
**RMS**	Rostral migration stream.
**TE**	Time of echo (one of the basic MRI/MRS pulse sequence parameters).
**T_1_w**	T_1_-weighted MRI.
**T_2_w**	T_2_-weighted MRI.
**T_2*_w**	T_2*_-weighted MRI.
**SVZ**	Subventricular zone.
**WM**	White matter.
