# Supplementary material for: Contribution of Magnetic Resonance Imaging Studies to the Understanding of Cerebral Malaria Pathogenesis
Source: Pathogens. 2024 Nov 27;13(12):1042. doi: 10.3390/pathogens13121042 (PMC11728472; doi:10.3390/pathogens13121042)
Supplement: Supplementary file 1 [file pathogens-13-01042-s001.zip › pathogens-3304628-supplementary.pdf]

Supplementary Table S1. MRI/MRS studies of ECM

REFERENCES	MURINE MALARIA MODEL	PRECLINICAL SCANNER FIELD STRENGTH, IMAGING TECHNIQUE	MRI FINDINGS	MRS FINDINGS	SIGNIFICANCE FOR THE PATHOLOGY
[1]	50 CBA/J females aged 8-10 wks (20 controls and 30 infected)<br><br>Infection with red blood cells parasitized with PbA<br><br>The mice were explored at mild (d4 p.i.) and severe (d7 p.i.) stages of CM	@ 4.7T and 11.75T<br><br>Anatomical MRI: T_2w MRI (SE) and T_2*w (GRE)<br><br>Contrast-enhanced MRI : T_1w MRI + Gd-DTPA (iv)<br><br>DWI (SE -EPI)<br><br>Perfusion MRI (ASL)<br><br>GRE-TOF MRA (11.75T)<br><br>^{1}H-MRS (PRESS) and ^{31}P-MRS (single pulse with a surface coil)	Severe stage of CM: Brain swelling, enlarged ventricles, brainstem engulfment, cerebellum crushing<br>Focal hyperintense lesions on T_2w-MRI<br>Focal hypointensities on T_2*w-MRI<br><br>Widespread enhancement of the parenchyma on T_1w MRI after Gd-DTPA injection<br><br>Decreased ADC in striatum and parietal cortex<br><br>Strong CBF reduction in cortex and striatum.<br>Reduced blood flow in arteries	Decreased NAA<br>Increased Glx<br>Increased lactate<br>Decrease in (PCr + β-ATP)/Pi<br>Decrease in cerebral pH	Massive ischemic brain edema is the main feature of CM and the cause of death<br><br>Mild and severe CM: Brain swelling, inflammatory disruption of the BBB, ventricle enlargement and ventriculitis<br><br>Severe CM: Massive vasogenic edema with cerebellum crushing and brainstem engulfment<br><br>Cytotoxic edema: Lesions in the white matter tracts and caudate putamen<br><br>Vascular/microvascular system: microhemorrhages, compression of cerebral arteries and reduced CBF due to ischemia<br><br>Ischemic metabolic profile: Neuronal damage and excitotoxicity

[2]	4 C57BL/6 mice aged 5-7 wks (sex not specified)<br><br>Infection with PbA (parasite stage not specified)<br><br>The mice were explored at d6 p.i.	@ 9.4T<br><br>Anatomical MRI: T_2w MRI (SE)<br><br>Perfusion MRI (ASL)<br><br>^{1}H-MRS (LASER)	No structural changes detected in anatomical MRI<br><br>Reduction of CBF in infected mice at the peak of the disease	Reduction of NAA/Cre ratio at the peak of the disease	<u>Vascular function</u>: impaired cerebral perfusion<br><br><u>Neurometabolism</u>: neuronal damage
[3]	30 BALB/c females aged 8-10 wks (10 controls, 20 infected)<br><br>The BALB/c strain is resistant to ECM but develop severe malarial anemia due to hyperparasitemia<br><br>Infection with red blood cells parasitized with PbA<br><br>The mice were explored at d8 and d15 (hyperparasitized mice) p.i.	@ 4.7T and 11.75T<br><br>Anatomical MRI : T_2w (SE) and T_2*w (GRE)<br><br>Contrast enhanced MRI: T_1 w + Gd-DTPA (iv)<br>DWI (SE-EPI)<br>Perfusion MRI (ASL)<br>GE-TOF MRA<br>^{1}H-MRS (PRESS) and ^{31}P-MRS<br>*Ex vivo* ^{1}H-MRS of whole brain extracts	<u>Exploration at d8:</u><br>No cerebral lesions on T_2w-MRI<br>No contrast-enhancement<br>No abnormal signal on T_2*w-MRI<br>No change in brain or ventricle volume<br>No change in ADC or CBF<br>No anomalies on angiography<br><br><u>Exploration at d15</u><br>Moderate but significant brain swelling<br><br>Significant increase in CBF	<u>Exploration at d8:</u><br>No metabolic anomaly on ^{1}H-MRS spectra<br><br><u>Exploration at d15:</u><br>Decrease of Cho (*in vivo* ^{1}H-MRS)<br>No anomaly detected with *in vivo* ^{31}P-MRS<br><br>Increase in glutamine, glycine, and alanine concentrations (*ex vivo* ^{1}H-MRS)<br>Decrease of acetate, aspartate, glycerophosphocholine, and *myo*-inositol (*ex vivo* ^{1}H-MRS)	Exploration at d8:<br>No anomaly detected at MRI<br><br>Exploration at d15 (anemic mice):<br>Moderate <u>brain swelling</u><br><br><u>Vascular/microvascular system</u>: increase in CBF<br><br><u>Neurometabolism</u>: hyper-parasitemia is associated with impaired choline-metabolism<br><br>*Ex vivo* ^{1}H-MRS of brain extracts indicate liver-related metabolic anomalies (hepatic encephalopathy)<br>Severe hyperparasitemia induces brain anomalies different to those caused by CM

[4]	3 C57BL/6 females per control and CM groups (age not specified)<br><br>Infection with red blood cells parasitized with PbA<br><br>The mice were explored at d5-7 p.i.	@ 7T<br><br>Anatomical MRI: T_2*w (GRE)<br><br>Contrast enhanced MRI:<br>1st CM group: T_1w + Gd-DTPA and<br>2nd CM group: T_2w + LIBS-MPIO (platelet-specific contrast agent)	Hyperintense areas on T_2w-MRI and on T_1w-MRI after injection with Gd-DTPA (d7)<br><br>Increase of T_2 on T_2-maps in the hippocampus (d7)<br><br>Hypointensities on T_2w-MRI after LIBS-MPIO injection at d5-6<br><br>The binding is enhanced after TNF injection in the brain		BBBD<br><br>Vascular/microvascular system: aggregation of activated endovascular platelets detectable before onset of clinical signs
[5]	21 CBA/J Females aged 8-10 wks (7 controls, 14 infected).<br><br>Infection with red blood cells parasitized with PbA<br><br>The mice were explored at early (d4-5 p.i.) and acute (d7-8 p.i.) stage of ECM	@ 11.75 T<br><br>Anatomical MRI: T_2w (SE), T_1w (3D GRE with strong T_2*w)	Early CM<br>T_2w-MRI hypointensities in the 2n and 5n at d4-5<br>Reduction of internerve distance<br>Crushing of 2n and 5n<br><br>Severe CM<br>Focal hyperintense parenchymal lesions and hypointensities on T_2w-MRI:<br>Alteration of external and internal capsules<br><br>Brain swelling and protrusion of the brainstem into the foramen magnum, cerebellum crushing<br><br>Discrete focal hemorrhages in cerebrum, cerebellum, brainstem and OB		Brain edema with cerebellum crushing and brainstem engulfment<br><br>Parenchymal lesions extending from the OB to the brainstem, WM and caudate-putamen<br><br>Newly described early lesions in CM: cranial nerve injury (2n and 5n) appears as the earliest anatomical sign of the disease, even before brain edema

[6]	C57BL/6 mice (post-mortem brains, PFA fixed), age not specified<br><br>Infection with PbA (parasite stage not specified)<br><br>The mice were explored at d6-8 p.i.	@ 11.75 T<br><br>Anatomical MRI: T_2*w (FLASH)<br><br>DWI (SE)	Dark spots in the OB at d6 post infection on T_2*w-MRI<br><br>Hypointense regions in OB at d6 on DWI		Identification of the OB as a vulnerable location for vascular leakage during ECM<br><br>The microbleedings in the OB are an early sign of CM<br>Olfaction loss might be a possible early sign of CM
[7]	21 C57BL/6 females aged 6-8 wks<br><br>Infection with PbA sporozoites<br><br>The mice were explored at d6-7 p.i.	@ 9.4T<br><br>Anatomical MRI: T_1w, T_2w (SE-RARE), T_2 relaxometry (MSME) and T_2*w FLASH)<br>TOF MRA<br>Contrast enhanced MRI: T_1w + Gd-DTPA and Gf-M<br>DWI (SE-EPI)	Multifocal hyperintense lesions on T_1w-MRI and T_2w-MRI<br><br>Early Gf-M leakage and Gd-DTPA extravasation on T_1w-MRI<br><br>Increased T_2 and ADC values		BBBD<br><br>Vasogenic edema<br><br>Vascular function: microhemorrhages confined to the OB<br><br>The BBBD and edema start in the OB and spread along the RMS posterior and deeper in the brain towards the DMS
[8]	50 C57BL/6 females aged 6-7 wks (38 controls, 12 infected)<br><br>Infection with red blood cells parasitized with PbA<br><br>The mice were explored on d5, d6, d8 and d12 p.i.	@ 9.4T<br><br>Anatomical MRI: T_2w<br>T_2 relaxometry (MSME)<br><br>Contrast enhanced MRI: T_1w + Gd-DTPA<br><br>DWI (SE-EPI)	Increased signal in the WM of the OB, CC and external capsule on T_2w-MRI<br><br>Increased ADC values in the OB<br>Reduced ADC values in the cerebellum<br><br>Extravasation of the contrast agent uptake in the OB, CC, external capsule and ventricles<br><br>The treatment with a glutamine antagonist on d6		Vasogenic edema in the OB and WM tracts<br><br>Cytotoxic edema in the cerebellum<br><br>Compression of cerebellar tissues maybe due to ischemic changes<br><br>BBBD in the OB and blood-CSF barrier disruption in the ventricles

			reduces the contrast enhancement and the ADC values		Treatment with a glutamine antagonist on d6 resolves edema and BBB disruption<br><br>Glutamate excitotoxicity plays a key role in BBB disruption and evolution of ECM<br><br>These are the first class of therapeutics to show efficacy after the onset of clinical signs
[9]	35 C57BL/6 J females aged 6-8 wks (12 controls, 23 infected)<br><br>Infection with PbA sporozoites<br><br>The mice were explored at d6-7 p.i.	@ 9.4T<br><br>Anatomical MRI: T_1w (Look-Locker), T_2w (SE), T_2*w (GRE)<br><br>Contrast enhanced MRI: T_1w + MPO-Gd<br>T_2*w + CLIO-NP<br><br>*Ex vivo* MRI: T_2*w (GRE)	MPO-Gd enhancement in the OB and along the RMS and DMS on T_1w-MRI<br><br>Focal hypointensities caused by CLIO-NP deposition in the same areas of Gd leakage on T_2*w-MRI<br><br>Leaking of MPO-Gd into the ventricular system on T_1w-MRI<br><br>Global brain volume increase on T_1w-MRI		<u>Inflammatory BBBD</u> in the OB and along the RMS and DMS and blood-CSF barrier disruption<br>correlating with disease severity and brain swelling<br><br>Severe <u>brain edema</u><br><br>Severe <u>vascular inflammation</u> mediated by the enzyme MPO in response to the parasite

[10]	32 C57BL/6 J females aged 6-8 wks<br><br>Infection with PbA sporozoites<br><br>The mice were explored at d6-12 p.i.	@ 9.4T<br>Anatomical MRI: T_2w (SE), T_2*w (Compensated GRE), and Multi GRE for R_2* mapping<br><br>Contrast-enhanced MRI: T_1w + Gd-DTPA<br><br>*Ex vivo* MRI: T_2*w (GRE)	Rostral BBBD with edema at the peak of infection<br><br>Microhemorrhages in OB		The severity of the disease at the peak of parasitemia correlates with the amount and size of microhemorrhages after edema resolution<br><br>Permanent CM lesions are worse in areas of most severe BBBD

[1] Abbreviations: 2n, optic nerves; 5n, trigeminal nerves; ADC, apparent diffusion coefficient; ASL, arterial spin labeling; BBB, blood brain barrier; BBBD, blood brain barrier disruption; CBF, cerebral blood flow; Cho: choline-containing compounds (mostly phosphocholine and glycerophosphocholine); CC, corpus callosum; CLIO, crosslinked iron oxides; Cre, creatine + phosphocreatine; CSF, cerebrospinal fluid; DMS, dorsal migratory stream; DWI, diffusion-weighted imaging; EPI, echoplanar imaging; FLASH, fast low angle shot magnetic resonance imaging; Gd-DTPA, gadopentetic acid; Gf-M, gadofluorine M; Glx, glutamate + glutamine; GRE, gradient echo ; Lac, lactate; LASER, localization by adiabatic selective refocusing; LIBS-MPIO, ligand-induced binding sites (of GPIIb/IIIa)-microparticles of iron oxide; MSME, multi-slice multi-echo; MRA, magnetic resonance angiography; NAA, *N*-acetylaspartate; OB, olfactory bulbs; PbA, *Plasmodium Berghei* ANKA; PCr, phosphocreatine; p.i., post inoculation; PFA, paraformaldehyde; PRESS, point resolved spectroscopy; RARE, rapid acquisition with relaxation enhancement; RMS, rostral migratory stream; SE, spin echo; T_1w, T_1-weighted imaging; T_2w, T_2-weighted imaging; TOF, time-of-flight; wk, week; WM, white matter.

Supplementary Table S2. MRI/MRS studies of HCM

REFERENCES	POPULATION (Age and country)	CLINICAL SCANNER, FIELD STRENGTH, IMAGING TECHNIQUE	MAIN FINDINGS	SIGNIFICANCE FOR THE PATHOLOGY
[11]	24 adults (mean age 27 years)<br>20 males 4 females<br>Imaged during acute phase of CM, at discharge and 6-18 months later<br>Thailand<br>Local	@ 0.2T<br>Anatomical MRI: T_1w, T_2w (DE)	Swelling of the brain during acute phase, compression of frontal horns and slight displacement of the brainstem in some cases<br><br>No hyperintensity in cerebral matter on T_2w-MRI	<u>Increased brain volume</u> that decreases after recovery and eventually returns to normal
[12]	3 cases:<br>13-year-old female (Cameroon)<br>30-year-old male (Niger)<br>23-year-old male (Thailand)<br>Imaged 36 hours and 4 weeks after the onset of illness/coma<br>Travelers (hospital in France)	@1.5T<br>Anatomical MRI: T_1w, T_2w, FLAIR<br>Contrast enhanced MRI: T_1w + contrast agent	Hemorrhagic cortical lesions<br><br>Focal and diffuse bilateral WM hyperintensities	<u>Parenchymal lesions</u>: focal and diffuse white matter lesions<br><u>Vascular function:</u> small cortical infarcts due to the blockage of capillaries
[13]	1 case: 87-year-old female (Haiti)<br>Imaged 7 days after onset of symptoms<br>Traveler (hospital in USA)	@1.5T<br>Anatomical MRI: T_1w, T_2w, FLAIR<br>Contrast enhanced MRI: T_1w + Gd-DTPA<br>DWI	Multiple foci of hyperintensity on periventricular and subcortical WM on T_2w and FLAIR MRI<br><br>Some hyperintense lesions on DWI with decreased ADC	<u>Parenchymal lesions</u>: multifocal lesions in the WM<br>Areas of <u>restricted diffusion</u> consistent with cytotoxic edema
[14]	10 adults (9 with uncomplicated malaria and 1 with CM)<br>Imaged on the day of admission, after clearance of parasitemia and 28 days later<br>Thailand	@3T<br>Anatomical MRI: 3D T_1w, FLAIR, T_2w<br>^{1}H-MRS: 2D-CSI	The patient with CM had a CSF Lac/Cre ratio 3x larger than the patients with uncomplicated malaria on 2D-CSI which decreased with parasitemia	<u>Neurometabolism</u>: CSF Lac/Cre increases in CM and decreases after treatment
[15]	3 adults<br>Imaged 24 hours after the onset of neurological symptoms	@1.5T	Multifocal hyperintensities in the bilateral periventricular WM, CC,	<u>Parenchymal lesions</u>: Focal abnormalities in WM, CC and

	22-30 years old<br>2 males + 1 female<br>India<br>Local	Anatomical MRI: T_1w, T_2w, FLAIR, T_2*w (GRE),<br>DWI	subcortical regions and thalami on T_2w-MRI and FLAIR-MRI	thalamus; and subcortical infarcts maybe due to capillary blockage
[16]	2 cases<br>24- and 32-year-old females<br>*P. falciparum* and *P. vivax*<br>Imaged during acute phase of the disease<br><br>India<br><br>Local	@1.5T<br>Anatomical MRI: T_1w, T_2w (SE), T_2w (FLAIR)<br>DWI	Diffuse brain swelling, disappearance of the sulcal spaces<br><br>Bilateral hyperintense lesions on basal ganglia, thalami, and superior cerebellum on T_2w-MRI<br><br>Hypointensities in thalami and cerebellum on T_1w-MRI<br><br>Hyperintensity and low ADC in basal ganglia on DWI	<u>Brain swelling</u><br><u>Parenchymal lesions</u>: lesions in basal ganglia, thalami, and cerebellum, that possibly represent infarcts<br><br><u>Restricted diffusion</u> in the basal ganglia, consistent with cytotoxic edema
[17]	4 cases<br>Imaged during acute phase of the disease<br>25 - 55 years old<br>3 males and 1 female<br>India<br>Local	Magnetic field not disclosed<br><br>Anatomical MRI: T_1w, T_2w, T_2*w (GRE)<br>DWI	Hypointensities in bilateral thalami on T_1w-MRI<br><br>Multiple foci of hyperintensities in thalami, brainstem, cerebellum corona radiata and hippocampus on T_2w-MRI<br><br>Blooming artifacts in thalami and brainstem in GRE-MRI<br><br>Restricted diffusion in thalami, brainstem, periaqueductal GM and cerebellum in DWI<br>(among other structures)	<u>Vascular function</u>: acute infarctions in the bilateral thalami<br><br><u>Acute hemorrhagic infarctions</u> in brainstem, thalami, cerebellum, cerebrum and hippocampus

[18]	152 children (120 retinopathy positive and 32 retinopathy negative)<br>Images within 12 hours of admission<br>48-55 months old<br><br>Retinopathy -: 59.4% females<br>Retinopathy +: 45.8% females<br>Malawi<br><br>Local	@0.35T<br>Anatomical MRI: T_1w (FLAIR), PD, T_2w (FLAIR), T_2w-FRFSE, T_2*w (GRE)<br><br>EPI-DWI	Increased cerebral volume, with loss of sulci and uncal herniation<br><br>Supratentorial cortical thickening<br><br>Hyperintensities in basal ganglia, cortex, WM, CC, cerebellum and thalami on T_2w-FRFSE MRI<br><br>Reduced diffusion in basal ganglia, cortex, subcortical WM and CC on DWI	Severe cerebral edema with uncal and/or cerebellar herniation<br><br>Cortical thickening<br><br>Parenchymal lesions: focal cortical and deep WM abnormalities, periventricular WM changes<br><br>Restricted diffusion in several brain structures<br>The basal ganglia are the most common area where abnormalities are observed
[19]	38 survivors of pediatric CM<br>Imaged 6–24 months post neurological deficits identification<br><br>10-102 months old<br><br>66% males<br><br>Malawi<br><br>Local	@0.35T<br>Anatomical MRI: T_1w, T_2w-FRFSE, FLAIR<br>DWI	Periventricular and subcortical WM changes on T_2w-MRI<br>Cerebral atrophy on T_2w-MRI<br>Focal cortical anomalies on FLAIR-MRI	Nearly 75% of pediatric CM survivors with neurological deficits present structural brain abnormalities 6-24 months after the disease<br><br>These abnormalities include: generalized atrophy associated with focal seizures during CM<br><br>Subcortical WM gliosis linked to an acute ICP increase during CM<br><br>Periventricular gliosis associated with brain ischemia during the disease
[20]	217 children (44 malarial retinopathy negative; 173 retinopathy positive)	@0.35T	Children with retinopathy + CM present more frequently an increased cerebral volume	CM with retinopathy:<br>Severe brain edema

	Imaged 13.7 ± 12.4 hours after admission and 20.8 ± 13.4 hours after coma onset (clinically stable)<br>51-57 months old<br>43-49% males<br><br>Malawi<br>Local	Anatomical MRI: T_1w (FLAIR), PD, T_2w (FLAIR), T_2w-FRFSE, T_2*w (GRE)<br><br>DWI	Hyperintensities in the WM, cortex, pons, basal ganglia, thalamus, CC, posterior fossa on T_2w-MRI<br><br>Abnormalities in the WM, cortex, basal ganglia, CC and posterior fossa on DWI	<u>Parenchymal abnormalities</u> in the cortex, WM, thalamus and posterior fossa<br><u>Abnormal diffusion</u> in posterior fossa and basal ganglia, predictive of retinopathy status<br><br>In survivors of CM, morbidity is associated with increased brain volume and cortical T_2 and DWI abnormalities
[21]	43 adults<br>Imaged at maximum 48 hours after admission<br><br>(≥16 years old)<br><br>81% of males<br><br>Bangladesh<br><br>Local	@0.3T<br>-Anatomical MRI: T_1w, T_2w (TSE), FLAIR (TSE), T_2* (GRE)<br><br>@1.5T<br>-Anatomical MRI: T_1w, T_2w (TSE), FLAIR (TSE), T_2*w (GRE)<br>-Contrast enhanced MRI: T_1 (SE) + dimeglumine gadopentetate<br>DWI<br>^{1}H-MRS (STEAM)	Mild, diffuse swelling in the supratentorial region and posterior fossa, with normal signal on T_2w/FLAIR-MRI and DWI<br>Focal hyperintensities in basal ganglia, pons, CC, cortex and cerebellum on T_2w/FLAIR-MRI in 26% of patients<br><br>Low ADC in 31 % of patients on DWI<br><br>Raised Cho/Cre and Lac/Cre in ^{1}H-MRS	Diffuse, mild <u>brain swelling</u> and possible cytotoxic edema<br><br><u>Focal parenchymal lesions</u>: ischemic lesions in basal ganglia, pons, CC, cortex and cerebellum<br><br><u>Diffusion abnormalities</u> in the cerebral cortex<br><br><u>Neurometabolism</u>: Raised Cho and Lac ratios in the parietal cortex<br><br>No obvious relation between MRI findings and disease severity or death
[22]	168 children with CM<br>> 5 months old<br>52% males<br>Malawi<br>Local	@0.35T<br>Anatomical MRI: T_1w (FSE), T_1w-FLAIR, PD, T_2 (FRFSE), T_2w-(FLAIR), T_2*w (GRE)<br>EPI-DWI	Severely increased brain volume<br><br>Decreased prepontine and postpontine CSF levels<br><br>Hyperintensity in WM, thalamus, cortex, posterior fossa, pons,	<u>Increased brain volume</u>, decreased CSF in the prepontine space and brainstem herniation<br><br><u>Parenchymal lesions</u>: supratentorial GM and thalamic lesions

			brainstem and basal ganglia on T_2w-MRI<br><br>Increased signal in supratentorial GM, posterior fossa, CC and basal ganglia on DWI<br><br>Focal cortical lesions	The severely increased brain volume and the raised ICP that it causes are the features with the strongest association with death in children with CM<br><br>Brain swelling is transient in survivors
[23]	11 patients:<br>5 adults (22-40 years old)<br>6 children (5-15 years old)<br>Imaged within 10 hours of admission and 48-72 h later<br><br>80% males<br><br>India<br><br>Local	@1.5T<br>Anatomical MRI: T_1w, T_2w, T_2-FLAIR (TSE)<br>Contrast enhanced MRI: T_1w (SE) + gadobenate dimeglumine for CBF, CBV and MTT calculation<br>SWI<br>DWI	Generalized cortical thickening and effacement of sulci on T_2w-MRI<br><br>Low ADC in the basal ganglia<br>Vascular engorgement in the basal ganglia<br><br>5 patients present discrete areas of DWI hyperintensity + decreased ADC, consistent with cytotoxic edema, especially in the basal nuclei<br>PERFUSION	<u>Increased brain volume</u><br><br><u>Cytotoxic edema</u><br><br><u>Vascular function</u>: vascular congestion in the basal nuclei<br>Both mechanisms of edema can happen at the same time in CM, in different parts of the brain → multifactorial mechanisms of edema in CM
[24]	221 child survivors of CM<br>Imaged at 1, 12 and 60 months after recovery<br>> 6 months of age<br>52.9% females<br>Malawi<br>Local	@ 0.35T<br><br>Detailed description of the MRI protocol missing	Focal or diffuse atrophy or gliosis<br>Increased periventricular signal on T_2w-MRI<br><br>Stroke<br><br>All these lesions were detected in survivors after 1 month and were related to more severe cognitive impairment	The <u>persistence of MRI abnormalities</u> after disease correlate with more significant long term neurodevelopmental delays (social, motor and language impairments)

[25]	16 children<br>Imaged within 24 hours of admission<br>Mean age 6.5 years old<br>55.2% females<br><br>Zambia<br><br>Local	@1.5T<br>Anatomical MRI: T_2w (SE), T_2w-FLAIR<br>Contrast enhanced MRI: T_1w + Gd-DTPA<br>SWI<br>DWI	Most of the patients had moderate/mild brain edema<br><br>Increased signal in the subcortical WM, cortex, pons, brainstem, CC and basal ganglia on T_2w-MRI<br><br>Restricted diffusion in the subcortical WM and basal ganglia<br><br>Decreased SWI signal along the superficial and deep venous systems	Moderate brain swelling<br><br>Restricted diffusion in the WM<br><br>Parenchymal lesions: basal ganglia<br>Vascular function: Lesions in subcortical brain regions sensitive to venous obstruction<br><br>Presence of blood products and hemozoin in these areas<br><br>This vascular congestion possibly contributes to the brain swelling in CM
[26]	1 case: 21-year-old male<br>Imaged on day 6 of illness and day 40 after treatment with quinine<br><br>Kenya<br><br>Traveler	Magnetic field not disclosed<br>Anatomical MRI: T_2w-FLAIR<br>MRA-TOF	Narrowing and dilatation in multiple cerebral arteries on MRA on the day 6 of illness that eventually disappeared<br><br>Mild diffuse cerebral atrophy on T_2w-MRI on day 40 of illness	Vascular function: reversible cerebral vasoconstriction syndrome
[27]	71 child survivors of CM<br>Imaged after 1 month of hospital discharge<br>≥ 6 months old<br>Malawi<br>Local	@0.35T<br>Anatomical MRI without contrast agent injection (detailed description of the MRI protocol missing)	One month after recovery, 60% of the cases present mild or severe atrophy and 55% present focal abnormalities in periventricular and subcortical WM, cortex, CC, basal ganglia, pons, brainstem and cerebellum	Atrophy and focal abnormalities persist in children 1 month after CM<br><br>These sequelae are associated with a worse cognitive and developmental outcome a year later
[28]	269 children with CM<br>Imaged within 12 hours of admission<br>Mean age: 51.4 months old	@0.35T<br>Anatomical MRI: T_2w<br>DWI	Increased DWI signal intensity and reduced ADC (cortex and	CM is associated with restricted diffusion in several brain areas

	Malawi<br>Local		basal ganglia) and WM (bilateral, subcortical and CC)	An isolated subcortical WM diffusion pattern is associated with less severity and better prognosis in pediatric CM
[29]	65 CM patients<br>27 children (5-8 years old)<br>38 adults (31-38 years old)<br>Imaged within 10 hours of admission<br>47 males, 16 females<br><br>India<br><br>Local	Clinical scanner @1.5T<br>Anatomical MR: T_1w (SE), T_2w (TSE), (FLAIR)<br>EPI-DWI	Pediatric CM<br>Larger brain volume and brainstem herniation on T_1w-MRI<br><br>High ADC at time of admission<br><br>Low ADC in the deep and subcortical WM at follow-up<br><br>Adult CM<br>Moderate or no brain swelling, with no brainstem herniation on T_1w-MRI<br><br>Low ADC in the basal ganglia on DWI	Age specific patterns in CM<br><br>Children: severe edema and brainstem herniation are the ultimate cause of death<br><br>Vasogenic edema during acute disease and mild cytotoxic pattern, related to hypoxia, at follow-up, mostly in WM<br><br>Adults: milder brain swelling and more pronounced focal cytotoxic edema in deep GM structures<br><br>Death is associated with severe hypoxia<br><br>Both types of edema are resolved in survivors
[10]	27 CM patients<br><br>India<br><br>Local	@1.5T<br>Anatomical MRI: T_1w, T_2w<br>SWI	Enlarged brains in children<br>Microhemorrhages at GM-WM junction, CC, basal ganglia and cerebellum	More severe edema in pediatric than in adult CM<br><br>Same amount of microhemorrhages in pediatric and adult CM, the frequency correlates with disease severity
[30]	1 case: 33-year-old male<br><br>South Africa	Magnetic field not disclosed<br>Anatomical MRI: T_2w (FLAIR)<br>SWI	Low ADC in basal ganglia, CC, hippocampi and frontoparietal gyri on DWI	Restricted diffusion indicates cytotoxic edema

	Traveler	DWI	Hyperintensities in globus pallidus and CC on T_2w-MRI and FLAIR<br><br>Diffuse microhemorrhages in basal ganglia, CC and WM on SWI	Edema, ischemia and microhemorrhages are in agreement with erythrocyte sequestration and disruption of the BBB
[31]	1 case: 12-year-old female<br><br>Guinea-Bissau	Magnetic field not disclosed<br>Anatomical MRI: T_2w<br>DWI	Hyperintensities on T_2w-MRI<br><br>DWI hypersignal and decreased ADC in the CC	Cytotoxic edema in the CC
[32]	2 cases<br>28-year- old female and 17-year- old female<br><br>Ghana and Sierra Leone<br><br>Travelers	Magnetic field not disclosed<br>Anatomical MRI: T_2w<br>SWI<br>DWI	Hyperintensities on T_2w-MRI and increased ADC in the subcortical WM and corona radiata<br><br>Decreased ADC in the CC, basal ganglia and hippocampus<br><br>Microhemorrhages in the subcortical WM, CC, basal ganglia and cerebellum	The disease is characterized by generalized brain swelling<br><br>Presence of both diffuse severe vasogenic edema in brain areas sensitive to hypoxia and cytotoxic edema secondary to ischemia in the subcortical WM<br><br>Persistent brain alterations that correlate with cognitive impairments

[1] Abbreviations: ADC, apparent diffusion coefficient; BBB, blood brain barrier; CBF, cerebral blood flow; CBV, cerebral blood volume, CC, corpus callosum; Cho: choline-containing compounds; Cre, creatine + phosphocreatine; CSI, Chemical Shift Imaging; CSF, cerebral spinal fluid; DE, dual echo; DWI, diffusion weighted imaging; EPI, echoplanar imaging; FLAIR, Fluid attenuated inversion recovery (T_2w contrast with high susceptibility to edema); FRFSE, Fast recovery fast spin echo; Gd-DTPA, gadopentetic acid; GM, grey matter; GRE, gradient echo ; ICP, intracranial pressure; Lac, lactate; MRA, magnetic resonance angiography; MTT, mean transit time; PD, proton density; SE, spin echo; STEAM, STimulated Echo Acquisition Mode; SWI, susceptibility-weighted imaging; T_1w, T_1-weighted imaging; T_2w, T_2-weighted imaging; TOF, time-of-flight; TSE, Turbo spin echo; WM, white matter

References

1. Penet, M.-F.; Viola, A.; Confort-Gouny, S.; Le Fur, Y.; Duhamel, G.; Kober, F.; Ibarrola, D.; Izquierdo, M.; Coltel, N.; Gharib, B.; et al. Imaging Experimental Cerebral Malaria *In Vivo* : Significant Role of Ischemic Brain Edema. *J. Neurosci.* **2005**, *25*, 7352–7358, doi:10.1523/JNEUROSCI.1002-05.2005.
2. Kennan, R.P.; Machado, F.S.; Lee, S.C.; Desruisseaux, M.S.; Wittner, M.; Tsuji, M.; Tanowitz, H.B. Reduced Cerebral Blood Flow and N-Acetyl Aspartate in a Murine Model of Cerebral Malaria. *Parasitol. Res.* **2005**, *96*, 302–307, doi:10.1007/s00436-005-1349-z.
3. Penet, M.-F.; Kober, F.; Confort-Gouny, S.; Le Fur, Y.; Dalmasso, C.; Coltel, N.; Liprandi, A.; Gulian, J.-M.; Grau, G.E.; Cozzone, P.J.; et al. Magnetic Resonance Spectroscopy Reveals an Impaired Brain Metabolic Profile in Mice Resistant to Cerebral Malaria Infected with Plasmodium Berghei ANKA. *J. Biol. Chem.* **2007**, *282*, 14505–14514, doi:10.1074/jbc.M608035200.
4. von zur Muhlen, C.; Sibson, N.R.; Peter, K.; Campbell, S.J.; Wilainam, P.; Grau, G.E.; Bode, C.; Choudhury, R.P.; Anthony, D.C. A Contrast Agent Recognizing Activated Platelets Reveals Murine Cerebral Malaria Pathology Undetectable by Conventional MRI. *J. Clin. Invest.* **2008**, *118*, 1198–1207, doi:10.1172/JCI33314.
5. Saggu, R.; Faille, D.; Grau, G.E.; Cozzone, P.J.; Viola, A. In the Eye of Experimental Cerebral Malaria. *Am. J. Pathol.* **2011**, *179*, 1104–1109, doi:10.1016/j.ajpath.2011.05.044.
6. Zhao, H.; Aoshi, T.; Kawai, S.; Mori, Y.; Konishi, A.; Ozkan, M.; Fujita, Y.; Haseda, Y.; Shimizu, M.; Kohyama, M.; et al. Olfactory Plays a Key Role in Spatiotemporal Pathogenesis of Cerebral Malaria. *Cell Host Microbe* **2014**, *15*, 551–563, doi:10.1016/j.chom.2014.04.008.
7. Hoffmann, A.; Pfeil, J.; Alfonso, J.; Kurz, F.T.; Sahm, F.; Heiland, S.; Monyer, H.; Bendszus, M.; Mueller, A.-K.; Helluy, X.; et al. Experimental Cerebral Malaria Spreads along the Rostral Migratory Stream. *PLOS Pathog.* **2016**, *12*, e1005470, doi:10.1371/journal.ppat.1005470.
8. Riggle, B.A.; Sinharay, S.; Schreiber-Stainthorp, W.; Munasinghe, J.P.; Maric, D.; Prchalova, E.; Slusher, B.S.; Powell, J.D.; Miller, L.H.; Pierce, S.K.; et al. MRI Demonstrates Glutamine Antagonist-Mediated Reversal of Cerebral Malaria Pathology in Mice. *Proc. Natl. Acad. Sci.* **2018**, *115*, E12024–E12033, doi:10.1073/pnas.1812909115.
9. Hoffmann, A.; Pfeil, J.; Mueller, A.-K.; Jin, J.; Deumelandt, K.; Helluy, X.; Wang, C.; Heiland, S.; Platten, M.; Chen, J.W.; et al. MRI of Iron Oxide Nanoparticles and Myeloperoxidase Activity Links Inflammation to Brain Edema in Experimental Cerebral Malaria. *Radiology* **2019**, *290*, 359–367, doi:10.1148/radiol.2018181051.
10. Jin, J.; Ba, M.A.; Wai, C.H.; Mohanty, S.; Sahu, P.K.; Pattnaik, R.; Pirpamer, L.; Fischer, M.; Heiland, S.; Lanzer, M.; et al. Transcellular Blood-Brain Barrier Disruption in Malaria-Induced Reversible Brain Edema. *Life Sci. Alliance* **2022**, *5*, e202201402, doi:10.26508/lsa.202201402.
11. Looareesuwan, S.; Wilairatana, P.; Krishna, S.; Kendall, B.; Vannaphan, S.; Viravan, C.; White, N.J. Magnetic Resonance Imaging of the Brain in Patients with Cerebral Malaria. *Clin. Infect. Dis.* **1995**, *21*, 300–309, doi:10.1093/clinids/21.2.300.
12. Cordoliani, Y.-S.; Sarrazin, J.-L.; Felten, D.; Caumes, E.; Leveque, C.; Fisch, A. MR of Cerebral Malaria. *AJNR Am. J. Neuroradiol.* **1998**, 4.
13. Sakai, O.; Barest, G.D. Diffusion-Weighted Imaging of Cerebral Malaria. *J. Neuroimaging* **2005**, *15*, 278–280, doi:10.1177/1051228405277341.
14. Tosti, C.L.; Petersen, E.T.; Laothamatas, J.; Golay, X.; Swaminathan, S.V.; Cauteren, M.V.; Murdoch, J.; Lekprasert, V.; Tangpukdee, N.; Krudsood, S.; et al. Cerebrospinal Fluid Lactate in P. Falciparum Malaria: Measurement by Chemical Shift Imaging at 3 Tesla. *Proc Intl Soc Mag Reson Med* **2007**, *15*, 1.
15. Yadav, P.; Sharma, R.; Kumar, S.; Kumar, U. Magnetic Resonance Features of Cerebral Malaria. *Acta Radiol.* **2008**, *49*, 566–569, doi:10.1080/02841850802020476.
16. Medhi, N.; Das, S.B.; Das, R.R.; Medhi, S.; Sarma, P.; Duwara, R.; Das, P.; Saikia, R. MRI Findings of Cerebral Malaria: A Report of Two Cases. *Neuroradiol. J.* **2009**, *22*, 407–412, doi:10.1177/197140090902200408.
17. Rasalkar, D.D.; Paunipagar, B.K.; Sanghvi, D.; Sonawane, B.D.; Loniker, P. Magnetic Resonance Imaging in Cerebral Malaria: A Report of Four Cases. *Br. J. Radiol.* **2011**, *84*, 380–385, doi:10.1259/bjr/85759874.
18. Potchen, M.J.; Kampondeni, S.D.; Seydel, K.B.; Birbeck, G.L.; Hammond, C.A.; Bradley, W.G.; DeMarco, J.K.; Glover, S.J.; Ugorji, J.O.; Latourette, M.T.; et al. Acute Brain MRI Findings in 120 Malawian Children with Cerebral Malaria: New Insights into an Ancient Disease. *AJNR Am. J. Neuroradiol.* **2012**, *33*, 1740–1746, doi:10.3174/ajnr.A3035.
19. Kampondeni, S.D.; Potchen, M.J.; Beare, N.A.V.; Seydel, K.B.; Glover, S.J.; Taylor, T.E.; Birbeck, G.L. MRI Findings in a Cohort of Brain Injured Survivors of Pediatric Cerebral Malaria. *Am. Soc. Trop. Med. Hyg.* **2013**, *88*, 542–546, doi:10.4269/ajtmh.12-0538.
20. Postels, D.G.; Li, C.; Birbeck, G.L.; Taylor, T.E.; Seydel, K.B.; Kampondeni, S.D.; Glover, S.J.; Potchen, M.J. Brain MRI of Children with Retinopathy-Negative Cerebral Malaria. *Am. Soc. Trop. Med. Hyg.* **2014**, *91*, 943–949, doi:10.4269/ajtmh.14-0216.
21. Maude, R.J.; Barkhof, F.; Hassan, M.U.; Ghose, A.; Hossain, A.; Abul Faiz, M.; Choudhury, E.; Rashid, R.; Sayeed, A.A.; Charunwatthana, P.; et al. Magnetic Resonance Imaging of the Brain in Adults with Severe Falciparum Malaria. *Malar. J.* **2014**, *13*, 177, doi:10.1186/1475-2875-13-177.

22. Seydel, K.B.; Kampondeni, S.D.; Valim, C.; Potchen, M.J.; Milner, D.A.; Muwalo, F.W.; Birbeck, G.L.; Bradley, W.G.; Fox, L.L.; Glover, S.J.; et al. Brain Swelling and Death in Children with Cerebral Malaria. *N. Engl. J. Med.* **2015**, *372*, 1126–1137, doi:10.1056/NEJMoa1400116.
23. Mohanty, S.; Benjamin, L.A.; Majhi, M.; Panda, P.; Kampondeni, S.; Sahu, P.K.; Mohanty, A.; Mahanta, K.C.; Pattnaik, R.; Mohanty, R.R.; et al. Magnetic Resonance Imaging of Cerebral Malaria Patients Reveals Distinct Pathogenetic Processes in Different Parts of the Brain. *mSphere* **2017**, *2*, e00193-17, doi:10.1128/mSphere.00193-17.
24. Brim, R.; Mboma, S.; Semrud-Clikeman, M.; Kampondeni, S.; Magen, J.; Taylor, T.; Langfitt, J. Cognitive Outcomes and Psychiatric Symptoms of Retinopathy-Positive Cerebral Malaria: Cohort Description and Baseline Results. *Am. Soc. Trop. Med. Hyg.* **2017**, *97*, 225–231, doi:10.4269/ajtmh.17-0020.
25. Potchen, M.J.; Kampondeni, S.D.; Seydel, K.B.; Haacke, E.M.; Sinyangwe, S.S.; Mwenechanya, M.; Glover, S.J.; Milner, D.A.; Zeli, E.; Hammond, C.A.; et al. 1.5 Tesla Magnetic Resonance Imaging to Investigate Potential Etiologies of Brain Swelling in Pediatric Cerebral Malaria. *Am. J. Trop. Med. Hyg.* **2018**, *98*, 497–504, doi:10.4269/ajtmh.17-0309.
26. Yamamoto, K.; Kato, Y.; Shinohara, K.; Kutsuna, S.; Takeshita, N.; Hayakawa, K.; Iwagami, M.; Kano, S.; Watanabe, S.; Ohmagari, N. Case Report: Reversible Cerebral Vasoconstriction Syndrome in Cerebral Malaria. *Am. J. Trop. Med. Hyg.* **2018**, *98*, 505–507, doi:10.4269/ajtmh.17-0665.
27. Langfitt, J.T.; McDermott, M.P.; Brim, R.; Mboma, S.; Potchen, M.J.; Kampondeni, S.D.; Seydel, K.B.; Semrud-Clikeman, M.; Taylor, T.E. Neurodevelopmental Impairments 1 Year After Cerebral Malaria. *Pediatrics* **2019**, *143*, e20181026, doi:10.1542/peds.2018-1026.
28. Moghaddam, S.M.; Birbeck, G.L.; Taylor, T.E.; Seydel, K.B.; Kampondeni, S.D.; Potchen, M.J. Diffusion-Weighted MR Imaging in a Prospective Cohort of Children with Cerebral Malaria Offers Insights into Pathophysiology and Prognosis. *AJNR Am. J. Neuroradiol.* **2019**, *40*, 1575–1580, doi:10.3174/ajnr.A6159.
29. Sahu, P.K.; Hoffmann, A.; Majhi, M.; Pattnaik, R.; Patterson, C.; Mahanta, K.C.; Mohanty, A.K.; Mohanty, R.R.; Joshi, S.; Mohanty, A.; et al. Brain Magnetic Resonance Imaging Reveals Different Courses of Disease in Pediatric and Adult Cerebral Malaria. *Clin. Infect. Dis.* **2021**, *73*, e2387–e2396, doi:10.1093/cid/ciaa1647.
30. Beltagi, A.E.; Elsotouhy, A.; Al-Warqi, A.; Aker, L.; Ahmed, M. Imaging Features of Fulminant Cerebral Malaria: A Case Report. *Radiol. Case Rep.* **2023**, *18*, 3642–3647, doi:10.1016/j.radcr.2023.06.066.
31. Sousa, A.; Silva, T.M.; Conceição, C.; Vieira, J.P.; Gouveia, C.; Varandas, L. Cerebral Malaria and Cytotoxic Lesions of the Corpus Callosum. *Pediatr. Infect. Dis. J.* **2023**, *42*, e358–e359, doi:10.1097/INF.0000000000003963.
32. Coughlan, C.; Jäger, H.R.; Brealey, D.; Carletti, F.; Hyare, H.; Pattnaik, R.; Sahu, P.K.; Mohanty, S.; Logan, S.; Hoffmann, A.; et al. Adult Cerebral Malaria: Acute and Subacute Imaging Findings, Long-Term Clinical Consequences. *Clin. Infect. Dis.* **2024**, *78*, 457–460, doi:10.1093/cid/ciad651.
